# Mast cell activation disease: a concise practical guide for diagnostic workup and therapeutic options

**DOI:** 10.1186/1756-8722-4-10

**Published:** 2011-03-22

**Authors:** Gerhard J Molderings, Stefan Brettner, Jürgen Homann, Lawrence B Afrin

**Affiliations:** 1 Institute of Human Genetics, University Hospital of Bonn, Sigmund-Freud-Str. 25, D-53127 Bonn, Germany; 2 Department of Oncology, Hematology and Palliative Care, Kreiskrankenhaus Waldbröl, Dr.-Goldenburgen-Str. 10, D-51545 Waldbröl, Germany; 3 Department of Internal Medicine, Evangelische Kliniken Bonn, Waldkrankenhaus, Waldstrasse 73, D-53177 Bonn, Germany; 4 Division of Hematology/Oncology, Medical University of South Carolina, Charleston, South Carolina, USA

## Abstract

Mast cell activation disease comprises disorders characterized by accumulation of genetically altered mast cells and/or abnormal release of these cells' mediators, affecting functions in potentially every organ system, often without causing abnormalities in routine laboratory or radiologic testing. In most cases of mast cell activation disease, diagnosis is possible by relatively non-invasive investigation. Effective therapy often consists simply of antihistamines and mast cell membrane-stabilising compounds supplemented with medications targeted at specific symptoms and complications. Mast cell activation disease is now appreciated to likely be considerably prevalent and thus should be considered routinely in the differential diagnosis of patients with chronic multisystem polymorbidity or patients in whom a definitively diagnosed major illness does not well account for the entirety of the patient's presentation.

## Introduction

The term *mast cell activation disease *(MCAD) denotes a collection of disorders characterized by (1) accumulation of pathological mast cells in potentially any or all organs and tissues and/or (2) aberrant release of variable subsets of mast cell mediators. A classification has been proposed which differentiates several types and subclasses of MCAD (Table 1). The traditionally recognized subclass termed *systemic mastocytosis *(SM) includes disorders characterized by certain pathological immunohistochemical and mutational findings (the WHO criteria; Table 2; [1,2]) which are divided into several subtypes (Table 1). On the other hand, *mast cell activation syndrome *(MCAS) presents a complex clinical picture of multiple mast cell mediator-induced symptoms, failure to meet the WHO criteria for diagnosis of SM, and exclusion of relevant differential diagnoses [1,3-5]. Symptoms observed in patients with MCAS are little, if any, different from those seen in patients with SM [6-8]. Patients present variable and often fluctuating patterns of symptoms (Table 3; [9-15]) which depend on the tissue responses to mast cell mediators released both spontaneously and in response to trigger stimuli.

**Table 1:** Classification of mast cell activation disease (modified from [2-4]).

*Mast cell activation disease (MCAD)*	
Mast cell activation syndrome (MCAS)	

Systemic mastocytosis (SM) defined by the WHO criteria	• Indolent systemic mastocytosis • Isolated bone marrow mastocytosis • Smoldering systemic mastocytosis • Systemic mastocytosis with an associated clonal hematologic non-mast cell lineage disease • Aggressive systemic mastocytosis

Mast cell leukemia (MCL)	

**Table 2:** Criteria proposed to define mast cell activation disease (for references, see text).

Criteria to define *mast cell activation syndrome*	WHO criteria to define *systemic mastocytosis*
**Major criteria**	**Major criterion**
1. Multifocal or disseminated dense infiltrates of mast cells in bone marrow biopsies and/or in sections of other extracutaneous organ(s) (e.g., gastrointestinal tract biopsies; CD117-, tryptase- and CD25-stained)	Multifocal dense infiltrates of mast cells (>15 mast cells in aggregates) in bone marrow biopsies and/or in sections of other extracutaneous organ(s) (CD117-, tryptase- and CD25-stained)
2. Unique constellation of clinical complaints as a result of a pathologically increased mast cell activity (mast cell mediator release syndrome)	
**Minor criteria**	**Minor criteria**
1. Mast cells in bone marrow or other extracutaneous organ(s) show an abnormal morphology (>25%) in bone marrow smears or in histologies	1. Mast cells in bone marrow or other extracutaneous organ(s) show an abnormal morphology (>25%) in bone marrow smears or in histologies
2. Mast cells in bone marrow express CD2 and/or CD25	2. Mast cells in bone marrow express CD2 and/or CD25
3. Detection of genetic changes in mast cells from blood, bone marrow or extracutaneous organs for which an impact on the state of activity of affected mast cells in terms of an increased activity has been proved.	3. c-kit mutation in tyrosine kinase at codon 816 in mast cells in extracutaneous organ(s)
4. Evidence of a pathologically increased release of mast cell mediators by determination of the content of	4. Serum total tryptase >20 ng/ml (does not apply in patients who have associated hematologic non-mast-cell lineage disease)
• tryptase in blood	
• N-methylhistamine in urine	
• heparin in blood	
• chromogranin A in blood	
• other mast cell-specific mediators (e.g., leukotrienes, prostaglandin D_2_)	

The diagnosis *mast cell activation syndrome *is made if both major criteria or the second criterion and at least one minor criterion are fulfilled. According to the WHO criteria [1], the diagnosis *systemic mastocytosis *is established if the major criterion and at least one minor criterion or at least three minor criteria are fulfilled.

**Table 3:** Frequent signs and clinical symptoms ascribed to episodic unregulated release of mast cell mediators (modified from [12]; further references therein; an exhaustive survey is given in [50]).

Signs and Symptoms	
***Abdominal***	abdominal pain, intestinal cramping and bloating, diarrhea and/or obstipation, nausea, non-cardiac chest pain, Helicobacter pylori-negative gastritis, malabsorption

***Oropharyngeal***	burning pain, aphthae

***Respiratory***	cough, asthma-like symptoms, dyspnea, rhinitis, sinusitis

***Ophthalmologic***	conjunctivitis, difficulty in focusing

***Hepatic***	splenomegaly, hyperbilirubinemia, elevation of liver transaminases, hypercholesterolemia

***Splenomegaly***	

***Lymphadenopathy***	

***Cardiovascular***	tachycardia, blood pressure irregularity (hypotension and/or hypertension), syncope, hot flush

***Neuropsychiatric***	headache, neuropathic pain, polyneuropathy, decreased attention span, difficulty in concentration, forgetfulness, anxiety, sleeplessness, organic brain syndrome, vertigo, lightheadedness, tinnitus

***Cutaneous***	urticaria pigmentosa, hives, efflorescences with/without pruritus, telangiectasia, flushing, angioedema

***Abnormal bleeding***	

***Musculoskeletal***	muscle pain, osteoporosis/osteopenia, bone pain, migratory arthritis

***Interstitial cystitis***	

***Constitutional***	fatigue, asthenia, fever, environmental sensitivities

A rare variant of MCAD is mast cell leukemia (MCL; Table 1). This aggressive mast cell neoplasm is defined by increased numbers of mast cells in bone marrow smears (≥20%) and by circulating mast cells (reviewed in [2]). Patients typically suffer from rapidly progressive organopathy involving the liver, bone marrow and other organs. The bone marrow typically shows a diffuse, dense infiltration with mast cells. In typical MCL, mast cells account for more than 10% of blood leukocytes. In a smaller group of patients, pancytopenia occurs and mast cells account for less than 10% (aleukemic variant of MCL). The prognosis in MCL is poor. Most patients survive less than 1 year and respond poorly to cytoreductive drugs or chemotherapy.

Mast cell activation disease in general has long been thought to be rare. However, although SM and MCL as defined by the WHO criteria are truly rare, recent findings suggest MCAS is a fairly common disorder. Evidence has been presented for a causal involvement of pathologically active mast cells not only in the pathogenesis of SM and MCAS but also in the etiology of idiopathic anaphylaxis [16-18], interstitial cystitis [19], some subsets of fibromyalgia [20,21] and some subsets of irritable bowel syndrome [22-24].

### Pathogenesis

Mutations in kinases (particularly in the tyrosine kinase Kit) and in enzymes and receptors (JAK2, PDGFRα, RASGRP4, Src-kinases, c-Cbl-encoded E3 ligase, histamine H4 receptor) which are crucially involved in the regulation of mast cell activity have been identified as necessary to establish a clonal mast cell population, but other abnormalities yet to be determined must be added for the development of a clinically symptomatic disease ([7,8,25,26]; further references therein). The observations that the same KIT mutation (e.g. D816V) can be associated with both good prognosis as well as progression to advanced disease [27] and that the D816V mutation has also been detected in healthy subjects [28] highlight the potential role of other factors in determining the progression/outcome of the disease. Recent findings suggest that the immunohistochemical and morphological alterations which constitute the WHO criteria for SM (formation of mast cell clusters; spindle-shaped morphology of mast cells; expression of CD25 on mast cells; Table 2) are causally related to and specific for the occurrence of a mutation in codon 816 of tyrosine kinase Kit in the affected mast cells [6,29-31]. Another aspect that limits the diagnostic value of this mutation is that during progression of SM the Kit mutant D816V may disappear ([32]; own unpublished observation). Taken together, the recent genetic findings suggest that the clinically different subtypes of MCAD (encompassing SM, MCL, and MCAS) should be more accurately regarded as varying presentations of a common generic root process of mast cell dysfunction than as distinct diseases [4,7,8,11].

### Clinical diagnostics

**Figure 1:**
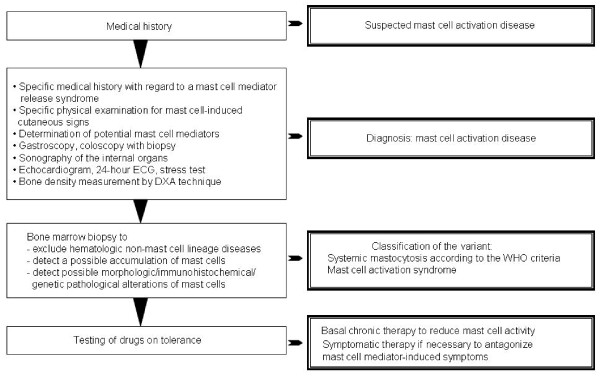
**Diagnostic algorithm**.

MCAD is first suspected on clinical grounds, based on recognition of compatible mast cell mediator-related symptoms and, in some, identification of typical skin lesions. The clinical presentation of MCAD is very diverse, since due to both the widespread distribution of mast cells and the great heterogeneity of aberrant mediator expression patterns, symptoms can occur in virtually all organs and tissues (Table 3). Moreover, symptoms often occur in a temporally staggered fashion, waxing and waning over years to decades. Symptoms often initially manifest during adolescence or even childhood or infancy but are recognized only in retrospect as MCAD-related. Clinical features and courses vary greatly and range from very indolent with normal life expectancy to highly aggressive with reduced survival times. Physical examination should include inspection for a large assortment of types of skin lesions, testing for dermatographism (Darier's sign), and palpating for hepatosplenomegaly and lymphadenopathy. A diagnostic algorithm is shown in Figure 1. Recognition of a mast cell mediator release syndrome, i.e. a pattern of symptoms caused by the unregulated increased release of mediators from mast cells, can be aided by use of a validated checklist [2,11,12,33] which lists the complaint complexes to be considered. In addition to the detection of the characteristic clinical constellation of findings, it must be investigated whether levels of the mast cell-specific mediators tryptase, histamine, and heparin are elevated in the blood, whether the excretion of the histamine metabolite methylhistamine into the urine is increased, and whether mast cell activity-related eosinophilia, basophilia or monocytosis in the blood can be observed. Other useful markers fairly specific to mast cells include serum chromogranin A (in the absence of cardiac and renal failure, neuroendocrine cancer, and proton pump inhibitor use) and serum and urinary leukotriene and prostaglandin isoforms (e.g., leukotriene E_4_, prostaglandin D_2_, and prostaglandin 9α,11βPGF_2_). Together with a characteristic clinical presentation, abnormal markers can be of diagnostic, therapeutic and prognostic relevance. However, it remains unsettled whether demonstration of an elevation of mast cell activity markers is absolutely necessary for diagnosis of MCAD because (1) many conditions (e.g., degrading enzymes, complexing molecules, tissue pH) may attenuate or impede spill-over of exocytosed mediators from tissues into the blood, (2) only a handful of the more than 60 releasable mast cell mediators can be detected by routine commercial techniques, and (3) mediator release syndrome may be due to an amplification cascade of basophil, eosinophil, and general leukocyte activation induced by liberation of only a few mast cell mediators [34] which, again, may not be detectable by present techniques.

When relevant differential diagnoses of a mast cell activation disease (Table 4) which may present mast cell mediator-induced symptoms by activation of normal mast cells (e.g., allergy) or as result of non-mast-cell-specific expression of mediators (e.g., neuroendocrine cancer) are excluded, the cause of the mast cell mediator release syndrome must lie in the uncontrolled increase in activity of pathologically altered mast cells. Patients with most types of MCAD often initially enjoy symptom-free intervals interspersed amongst symptomatic periods. Over time, symptom-free intervals shorten, and finally symptoms become chronic with intensity which fluctuates but with an overall trend toward steadily increasing intensity. Following the proposed revised diagnostic criteria (Table 2; [3-5,9,35]), MCAD is diagnosed if either both major criteria or one major criterion and at least one minor criterion are met. After clinical diagnosis, a bone marrow biopsy is usually recommended because based on current information it cannot be predicted whether the genetic alterations inducing pathological mast cell activity in affected mast cells have not also induced disturbances in hematopoietic non-mast cell lineages. SM due to codon 816 mutations has been shown to be associated with myeloid neoplasms (and, less frequently, with B-cell neoplasms) frequently enough to warrant routine marrow biopsy when SM is suspected (e.g., serum tryptase elevation per the WHO criteria, frequent unprovoked anaphylactoid events). The frequency of discovery of associated hematologic neoplasms on marrow biopsy at the time of diagnosis of MCAS remains unclear but in our experience appears very low. However, a byproduct of marrow biopsy is that immunohistochemical analysis of the specimen may permit the classification of the mast cell activation disease as SM defined by the WHO criteria or as MCAS (Table 2). In this context, it has to be considered that due to the typically patchy distribution of mast cell infiltration in the bones a single marrow biopsy fails to find systemic mastocytosis in the marrow approximately one-sixth of the time [36].

**Table 4:** Diseases which should be considered as differential diagnoses of mast cell activation disease, since they may mimick or may be associated with mast cell activation (diagnostic procedure of choice in parentheses).

*Endocrinologic disorders*	Diabetes mellitus (laboratory determination) Pancreatic endocrine tumours (gastrinoma, insulinoma, glucagonoma, somatostatinoma, VIPoma; laboratory determination, medical history) Porphyria (laboratory determination) Disorders of the thyroid gland (laboratory determination) Morbus Fabry (clinical picture, molecular genetic investigation)
***Gastrointestinal disorders***	Helicobacter-positive gastritis (gastroscopy, biopsy) Infectious enteritis (stool examination) Eosinophilic gastroenteritis (endoscopy, biopsy) Parasitic infections (stool examination) Inflammatory bowel disease (endoscopy, biopsy) Celiac disease (endoscopy, biopsy, laboratory determination) Primary lactose intolerance (molecular genetic investigation) Microscopic colitis (endoscopy, biopsy) Amyloidosis (endoscopy, biopsy) Intestinal obstructions by adhesions, volvulus and other reasons (medical history, imaging methods, laparoscopy) Hepatitis (laboratory determination) Cholelithiasis (imaging methods) Hereditary hyperbilirubinemia (laboratory determination)

***Immunological/neoplastic diseases***	Carcinoid tumour (medical history, laboratory determination) Pheochromocytoma (medical history, laboratory determination) Primary gastrointestinal allergy (medical history) Hypereosinophilic syndrome (laboratory determination) Hereditary angioedema (medical history, laboratory determination) Vasculitis (medical history, laboratory determination) Intestinal lymphoma (imaging methods)

An aggressive course of MCAD is characterized and defined by organopathy caused by pathologic infiltration of various organs by neoplastic mast cells inducing an impairment of organ function. Organopathy due to mast cell infiltration is indicated by findings termed *C-findings*: (1) significant cytopenia(s); (2) hepatomegaly with impairment of liver function due to mast cell infiltration, often with ascites; (3) splenomegaly with hypersplenism; (4) malabsorption with hypoalbuminemia and weight loss; (5) life-threatening impairment of organ function in other organ systems; (6) osteolyses and/or severe osteoporosis with pathologic fractures. Urticaria pigmentosa-like skin lesions are usually absent. In contrast to MCL, the bone marrow smear shows fewer than 20% mast cells (reviewed in [2]). Mast cell infiltration with organomegaly but without end organ dysfunction (hepatomegaly, splenomegaly, lymphadenopathy, bone marrow alterations) is a *B-finding *and may occur in a subvariant of SM (smoldering SM) with high mast cell burden.

### Treatment of mast cell activation diseases

The cornerstone of therapy is avoidance of identifiable triggers for mast cell degranulation such as animal venoms, extremes of temperature, mechanical irritation, alcohol, or medications (e.g., aspirin, radiocontrast agents, certain anesthetic agents). Individual patients may have variable tolerance patterns and avoidance lists, but it also is not uncommon to have no identifiable, reliable triggers.

Drug treatment of MCAD patients is highly individualized. Curative therapies are not avail-able, and each MCAD patient should be treated in accordance with his symptoms and complications. Irrespective of the specific clinical presentation of MCAD, evidence-based therapy consists of trigger avoidance, antihistamines, and mast cell membrane-stabilising compounds (basic therapy, Table 5) supplemented as needed by medications targeting individual mast cell mediator-induced symptoms or complications (symptomatic therapy, Table 5). First hints of success with any given therapy are usually seen within 4 weeks once suitable dosing has been achieved Multiple simultaneous changes in the medication regimen are discouraged since such can confound identification of the specific therapy responsible for a given improvement (or deterioration). Ineffective or harmful agents should be stopped promptly. If symptoms are resistant to therapy, as a next therapeutic step toward reducing mast cell activity and thereby decreasing mediator release, treatment with prednisone, ciclosporine (cyclosporine A), low dose methotrexate or azathioprine can be considered. Recently, anti-IgE treatment with the humanized murine monoclonal antibody omalizumab has alleviated high intensity symptoms of MCAD [37]. Since treatment with omalizumab has an acceptable risk-benefit profile, it should be considered in cases of MCAD resistant to evidence-based therapy. Recently, molecularly targeted therapy by tyrosine kinase inhibitors such as imatinib mesylate, dasatinib and midostaurin has been investigated. As with all drugs used in therapy of MCAD, their therapeutic success seems to be strongly dependent on the individual patient. In formal studies in SM patients, although the kinase inhibitors reduced mast cell burden as reflected by histological normalization in bone marrow and improved laboratory surrogate markers, at best only partial improvement of mediator-related symptoms was achieved [38-41]. However, in some case reports, imatinib and dasatinib have been significantly effective at relieving symptoms. In spite of potential significant adverse effects of these drugs, a therapeutic trial may be justified in individual cases at an early stage. Given that PI3K/AKT/mTOR is one of the downstream signalling pathways upregulated by activated Kit, in theory mTOR inhibitors (e.g., sirolimus, temsirolimus, everolimus) may have utility in MCAD, but to date the one trial of this notion (everolimus in SM) showed no significant clinical activity [42].

**Table 5:** Treatment options for mast cell activation disease.

**Basic therapy **(continuous oral combination therapy to reduce mast cell activity)	• H_1_-histamine receptor antagonist (to block activating H_1_-histamine receptors on mast cells; to antagonize H_1_-histamine receptor-mediated symptoms) • H_2_- histamine receptor antagonist (to block activating H_2_-histamine receptors on mast cells; to antagonize H_2_-histamine receptor-mediated symptoms) • Cromolyn sodium (stabilising mast cells) • Slow-release Vitamin C (increased degradation of histamine; inhibition of mast cell degranulation; not more than 750 mg/day) • If necessary, ketotifen to stabilise mast cells and to block activating H_1_-histamine receptors on mast cells
**Symptomatic treatment options **(orally as needed)	• ***Headache***⇒ paracetamol; metamizole; flupirtine • ***Diarrhea***⇒ colestyramine; nystatin; montelukast; 5-HT_3 _receptor inhibitors (eg. ondansetron); incremental doses (50-350 mg/day; extreme caution because of the possibility to induce mast cell degranulation) of acetylsalicylic acid; (in steps test each drug for 5 days until improvement of diarrhea) • ***Colicky abdominal pain***due to distinct meteorism ⇒ metamizole; butylscopolamine • ***Nausea***⇒ metoclopramide; dimenhydrinate; 5-HT_3 _receptor inhibitors; icatibant • ***Respiratory symptoms***(mainly increased production of viscous mucus and obstruction with compulsive throat clearing) ⇒ montelukast; urgent: short-acting ß-sympathomimetic • ***Gastric complaints***⇒ proton pump inhibitors (de-escalating dose finding) • ***Osteoporosis, osteolysis, bone pain***⇒ biphosphonates ([51]; vitamin D plus calcium application is second-line treatment in MCAD patients because of limited reported success and an increased risk for developing kidney and ureter stones; [52]) • ***Non-cardiac chest pain***⇒ when needed, additional dose of a H_2_-histamine receptor antagonist; also, proton pump inhibitors for proven gastroesophageal reflux • ***Tachycardia***⇒ verapamil; AT1-receptor antagonists; ivabradin • ***Neuropathic pain and paresthesia***⇒ α-lipoic acid • ***Interstitial cystitis***⇒ pentosan, amphetamines • ***Sleep-onset insomnia/sleep-maintenance insomnia***⇒ triazolam/oxazepam • ***Conjunctivitis***⇒ exclusion of a secondary disease; otherwise preservative-free eye drops with glucocorticoids for brief courses • ***Hypercholesterolemia***⇒ (does not depend on the composition of the diet) therapeutic trial with HMG-CoA reductase inhibitors (frequently ineffective) • ***Elevated prostaglandin levels, persistant flushing***⇒ incremental doses of acetylsalicylic acid (50-350 mg/day; extreme caution because of the possibility to induce mast cell degranulation)

All drugs should be tested for tolerance in a low single dose before therapeutic use, if their tolerance in the patient is not known from an earlier application.

A difficult situation is the occurrence of life-threatening anaphylaxis in patients with MCAD. If anaphylaxis is provoked by a known allergen, especially hymenoptera venom, immunotherapy should be considered with recognition of potential risks [43-45]. In case of repeated life-threatening anaphylactoid episodes, the self-administration of epinephrine on demand has been recommended as an appropriate approach.

In patients with high-grade variants of MCAD (presence of C-findings) and a progressive clini-cal course, cytoreductive drugs are recommended and are prescribed together with anti-mediator-type drugs [46,47]. Potential therapeutic options are interferon-α and 2-chlorodeoxyadenosine (2-CdA, cladribine). Interferon-α is frequently combined with prednisone and is commonly used as first-line cytoreductive therapy for aggressive SM. It ameliorates SM-related organopathy in a proportion of cases but is associated with considerable adverse effects (e.g., flu-like symptoms, myelosuppression, depression, hypothyroidism), which may limit its use in MCAD [48,49]. PEGylated interferon-α has been shown to be as efficacious as, and less toxic than the non-PEGylated form in some chronic myeloproliferative diseases, but it has not been specifically studied in MCAD. 2-Chlorodeoxyadenosine (2-CdA) is generally reserved for last choice treatment of patients with aggressive SM who are either refractory or intolerant to interferon-α. Potential toxicities of 2-CdA include significant and potentially prolonged myelosuppression and lymphopenia with increased risk of opportunistic infections. Patients who fail interferon-α and 2-CdA therapy are candidates for experimental drugs. However, such therapeutic maneuvers and their potential beneficial effects have to be balanced against the long-term risk and serious side effects of these therapies (often immunosuppressive or/and mutagenic). Polychemotherapy including intensive induction regimens of the kind used in treating acute myeloid leukemia, as well as high-dose therapy with stem cell rescue, represent investigational approaches restricted to rare, selected patients. A variety of other agents have been reported to have in-vitro activity against at least some MCAD-associated mutations [3] and may have a future role in the treatment of this disease.

No tools yet exist to predict which specific therapeutic regimen will be optimal for the individual MCAD patient. However, especially in non-aggressive disease (comprising the great majority of patients), at least partial improvement is usually attainable with one regimen or another, and thus the practitioner is obligated to persist with therapeutic trials until no options remain. Finally, although clinical trials in MCAD are rare, enrolment in such must be a priority.

## Conclusions

MCAD comprises disorders affecting functions in potentially every organ system by abnormal release of mediators from and/or accumulation of genetically altered mast cells. There is evidence that MCAD is a disorder with considerable prevalence and thus should be considered routinely in the differential diagnosis of patients with chronic multisystem polymorbidity of unknown cause. In most cases of MCAD, diagnosis is possible by relatively non-invasive investigation. Effective therapy often consists simply of antihistamines and mast cell membrane-stabilising compounds supplemented with medications targeted at specific symptoms and complications.

## Competing interests

The authors declare that they have no competing interests.

## Authors' contributions

All authors have equally contributed to draft the manuscript. All authors read and approved the final manuscript.
